# Hysteresis Modeling of Magnetic Shape Memory Alloy Actuator Based on Krasnosel'skii-Pokrovskii Model

**DOI:** 10.1155/2013/865176

**Published:** 2013-04-28

**Authors:** Miaolei Zhou, Shoubin Wang, Wei Gao

**Affiliations:** College of Communication Engineering, Jilin University, Changchun 130022, China

## Abstract

As a new type of intelligent material, magnetically shape memory alloy (MSMA) has a good performance in its applications in the actuator manufacturing. Compared with traditional actuators, MSMA actuator has the advantages as fast response and large deformation; however, the hysteresis nonlinearity of the MSMA actuator restricts its further improving of control precision. In this paper, an improved Krasnosel'skii-Pokrovskii (KP) model is used to establish the hysteresis model of MSMA actuator. To identify the weighting parameters of the KP operators, an improved gradient correction algorithm and a variable step-size recursive least square estimation algorithm are proposed in this paper. In order to demonstrate the validity of the proposed modeling approach, simulation experiments are performed, simulations with improved gradient correction algorithm and variable step-size recursive least square estimation algorithm are studied, respectively. Simulation results of both identification algorithms demonstrate that the proposed modeling approach in this paper can establish an effective and accurate hysteresis model for MSMA actuator, and it provides a foundation for improving the control precision of MSMA actuator.

## 1. Introduction

Hysteresis phenomena are widely exhibiting between input and output response of smart material actuators; MSMA actuator is a new kind smart material actuator which has more advantages than other smart material actuators: rapid frequency response, high control precision, and high deformation rate [1–3], but hysteresis between its input and output displacement is a barrier which reduces the control precision and restricts the further application of MSMA actuator. Therefore, in order to improve the position control accuracy of MSMA actuator, it is necessary to mitigate the effects of hysteresis. Many approaches have been proposed to mitigate the effects of hysteresis, and up until now, establishing the hysteresis model of smart material actuators is the most popular solution to this problem [4–8].

Preisach-type hysteresis models, such as Preisach model [9–11], Prandtl-Ishlinskii (PI) model [12, 13], and KP model [14, 15] are the most popular ones which have been widely used in modern control techniques to mitigate the effects of unknown hysteresis. In this paper, a Preisach-type hysteresis model—modified KP model is used to describe the hysteresis of MSMA actuator. KP model can be seen as an integral of a weighting function and KP operators; compared with other Preisach-type hysteresis models, KP model has the advantage as it can describe more complex hysteresis and behave well for offline identification. Improved gradient correction algorithm and variable step-size recursive least square estimation algorithm are applied to identify the weighting parameters, and simulation experiments are investigated in this paper to verify the validity of the proposed method.

## 2. Structure of the Hysteresis Model of MSMA Actuator Based on KP Model

**Figure 1:**
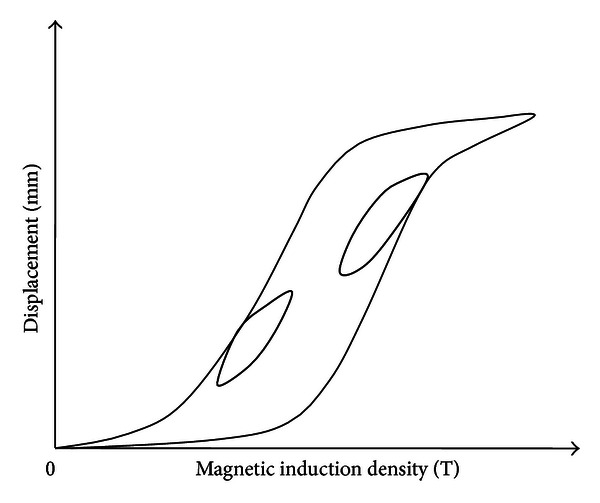
Major hysteresis loop and inner hysteresis loops of MSMA actuator.

The schematic diagram of hysteresis between input magnetic induction density and output displacement of MSMA actuator is shown in Figure 1. The hysteresis makes it difficult to get high control accuracy in the MSMA actuator-based system, and in this section, the structure of the proposed hysteresis model is described.

**Figure 2:**
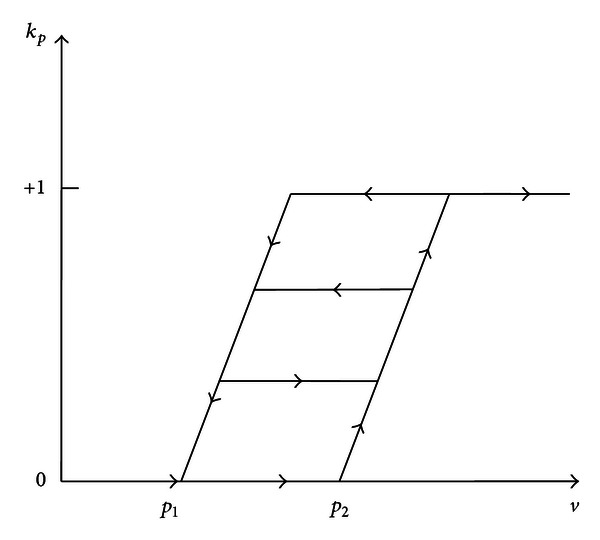
The modified KP operator.

The modified KP operator is shown in Figure 2; different from the traditional KP operator, the improved KP operator has the output range [0, +1], which is because the output of MSMA actuator is unidirectional [16].

**Figure 3:**
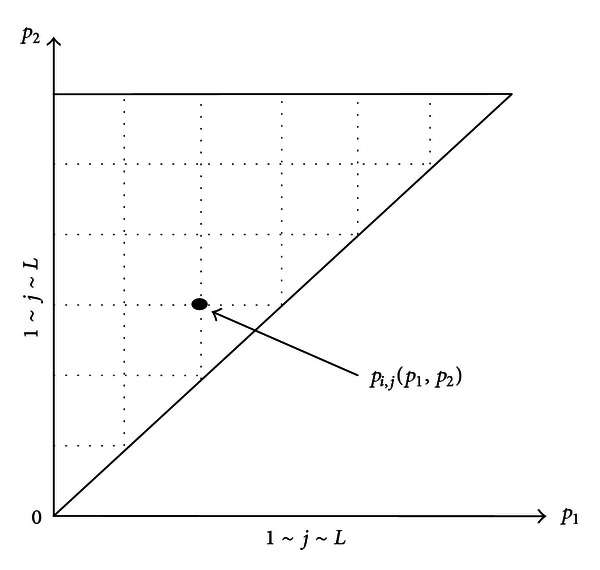
Preisach plane.

It can be seen from Figures 1 and 2 that the KP operator has some similar characters with the hysteresis loop of MSMA actuator. The KP model proposed in this paper can be expressed as an integral of the KP operators over a specific domain

(1) $$\begin{array}{c} u\left(t\right)=H\left[v\right]\left(t\right)=\int_{P}^{}k_{p}\left[v,\xi_{p}\right]\left(t\right)\mu \left(p\right)dp, \end{array}$$

where *v*(*t*) and *u*(*t*) are the input and output, respectively, *H* is the operator which can transform *v*(*t*) to *u*(*t*), and *P* is the Preisach plane which is a triangular domain as Figure 3 shows, and *P* = {*p*(*p*_1_, *p*_2_) ∈ *R* ^2^ : *v*_max⁡_ ≥ *p*_2_ ≥ *p*_1_ ≥ 0}, *k*_*p*_ is the KP operator, *μ*(*p*) is the weighting function to scale the output of the KP operator, and *ξ*_*p*_ is the historical extreme values of the KP operator.

**Figure 4:**
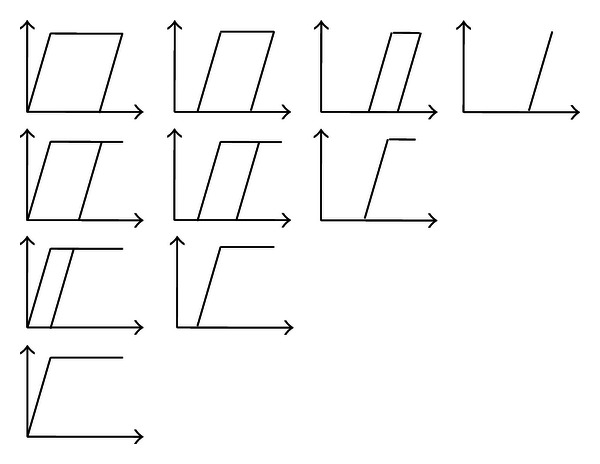
Schematic diagram of discrete KP model.

By dividing the Preisach plane into a mesh grid of *L* × *L* as shown in Figure 3, each point *p*_*i*,*j*_ in the Preisach plane represents a KP operator with its weighting value *μ*(*p*_*i*,*j*_) as shown in Figure 4. That means that there are *N* = *L* × (*L* + 1)/2 KP operators in the KP model. An approximation of the integral KP model can be gotten as a parallel connection of a number of weighted KP operators

(2) $$\begin{array}{c} u\left(t\right)=H\left[v\right]\left(t\right)=\sum_{j=1}^{L}\;\;\;\sum_{i=1}^{j}k_{p_{i,j}}\left[v,\xi_{p_{i,j}}\right]\mu \left(p_{i,j}\right). \end{array}$$

The function of KP operator *k*_*pi*,*j*_[*v*, *ξ*_*pi*,*j*_] is given in (3)

(3) $$\begin{array}{c} k_{p_{i,j}}\left[v,\xi_{p_{i,j}}\right]\left(t\right)=\{\begin{array}{cc} \max\left\{\xi_{p_{i,j}}\left(t\right),r\left(v\left(t\right)-p_{2}\right)\right\} & \dot{v}\geq 0\\ \min\left\{\xi_{p_{i,j}}\left(t\right),r\left(v\left(t\right)-p_{1}\right)\right\} & \dot{v}\leq 0. \end{array} \end{array}$$

The function of *ξ*_*p*_ is

(4) $$\begin{array}{c} \xi_{p_{i,j}}\left(t\right)=\{\begin{array}{cc} 0 & \cdots \\ k_{p_{i,j}}\left[v\left(t\right),\xi_{p_{i,j}}\left(t_{i-1}\right)\right]\left(t\right) & \cdots \\ \xi_{p_{i,j}}\left(t_{i-1}\right) & \cdots \end{array}\\ t=t_{0},\\ t=t_{i}>t_{i-1},\;\mathrm{sign}\left(\dot{v}\left(t^{+}\right)\right)=-\mathrm{sign}\left(\dot{v}\left(t^{-}\right)\right),\\ t_{i}\geq t>t_{i-1},\;\mathrm{sign}\left(\dot{v}\left(t^{+}\right)\right)=\mathrm{sign}\left(\dot{v}\left(t^{-}\right)\right), \end{array}$$

where the ridge function for two boundaries of KP operator can be expressed by

(5) $$\begin{array}{c} r\left(x\right)=\{\begin{array}{cc} 0 & x<0\\ \frac{x}{a} & 0\leq x\leq a\\ 1 & x>a, \end{array} \end{array}$$

where *a* = 1/(*L* − 1).

## 3. Identification Methods of the Hysteresis Model

From (2), it can be known that the KP model can be expressed by a parallel connection of a number of weighted KP operators like a neural network; that means that there are *N* = *L* × (*L* + 1)/2 weighting parameters needed to be identified, and the weighting parameters identification can affect the modeling accuracy directly. In this paper, two identification methods are adopted to identify the established KP model: improved gradient correction algorithm and variable step-size recursive least square estimation algorithm.

### 3.1. The Improved Gradient Correction Algorithm

The recurrence formulas of gradient correction parameter estimation for deterministic problems are as follows [17]:

(6) $$\begin{array}{c} \hat{\theta }\left(t+1\right)=\hat{\theta }\left(t\right)+R\left(t\right)h\left(t\right)\varepsilon \left(t\right),\\ \varepsilon \left(t\right)=y\left(t\right)-h^{T}\left(t\right)\hat{\theta }\left(t\right), \end{array}$$

where $\hat{\theta }(t)$ is the parameters evaluation vector at time *t*, *R*(*t*) is the weight matrix, *h*(*t*) is the vector of the KP operators' values at time *t*, and *y*(*t*) is the actual output at time *t*.

Equation (6) is the traditional recurrence formula of gradient correction parameter estimation for deterministic problems; however, with this identification method, the modeling accuracy is not satisfactory, and therefore, an improved gradient correction algorithm is proposed in this paper. The algorithm of the improved gradient correction algorithm is as follows

(1) Set an expectation error *E*. Assume that there are NU sets of data which are used to do the identification.
(2) Assign a zero vector to the initial value $\hat{\theta }(1)$, and assign a zero vector to *ε*(1). Set CN = 1, where CN is the cycle number.
  (i) If CN ≥ MCN, go to Step (3), else set *t* = 1 and continue, where *MCN* is a maximum cycle number used to avoid the endless loop.
  (ii) Get the modeling error $\varepsilon (t)=y(t)-h^{T}(t)\hat{\theta }(t)$, $\hat{\theta }(t+1)=\hat{\theta }(t)+R(t)h(t)\varepsilon (t)$, if *Max*⁡[|*ε*(*t*)|] ≤ *E*, *t* = *t* + 1, go to Step (3), and else continue. In this step, the weight matrix *R*(*t*) = I/[*e* ^*i*^0.19^^ × ∑_*i*=1_^*N*^ *h* ^2^(*i*, *t*)], and *I* is a unit matrix.
  (iii) If *t* = NU, set CN = CN + 1, *t* = *t* + 1, and $\hat{\theta }(1)=\hat{\theta }(t)$ and back to Step (i), else *t* = *t* + 1 and back to Step (ii).
(3) Set $c=\hat{\theta }(t)$, and *c* is the weighting parameters gotten after the identification.

In the proposed improved gradient correction algorithm above, the expectation error *E* and the maximum cycle number MCN can be set according to the requirements.

### 3.2. Variable Step-Size Recursive Least Square Estimation Algorithm

In the system identification methods, least square method is the most widely used one, the recursive least square method is especially popular. In the recursive least square method, the parameters evaluation is updated at every time when a new set of observation data is gotten. In order to reduce the computations in the identification process, a variable step-size recursive least square estimation algorithm is adopted. The algorithm principle of the variable step-size recursive least square estimation algorithm is as follows [18].

Assume that the formula of least square estimation algorithm is

(7) $$\begin{array}{c} {\hat{\theta }}_{\text{WLS}}={\left[\sum_{i=1}^{N}\Lambda \left(i\right)h\left(i\right)h^{T}\left(i\right)\right]}^{-1}\left[\sum_{i=1}^{N}\Lambda \left(i\right)h\left(i\right)y\left(i\right)\right], \end{array}$$

where ${\hat{\theta }}_{\text{WLS}}$ is weighting parameters gotten after the identification, Λ(*i*) is the weighted factor, *h*(*i*) is the values of the KP operators, and *y*(*i*) is the actual output. Assume that *P* ^−1^(*k*) = ∑_*i*=1_^*k*^Λ(*i*)*h*(*i*)*h* ^*T*^(*i*), *P* ^−1^(*k* − *l*) = ∑_*i*=1_^*k*−*l*^Λ(*i*)*h*(*i*)*h* ^*T*^(*i*), and *l* is the step-size which is an integer greater than 0, then there is

(8) $$\begin{array}{c} P^{-1}\left(k\right)=P^{-1}\left(k-l\right)+H_{k,l}^{T}\Lambda_{k,l}H_{k,l}, \end{array}$$

where *H*_*k*,*l*_ = [*h*(*k* + 1 − *l*), *h*(*k* + 2 − *l*),…, *h*(*k*)]^*T*^ and Λ_*k*,*l*_ = diag⁡[Λ(*k* + 1 − *l*), Λ(*k* + 2 − *l*),…, Λ(*k*)], and according to (7), there is

(9) $$\begin{array}{c} \hat{\theta }\left(k-l\right)=P\left(k-l\right)\left[\sum_{i=1}^{k-l}\Lambda \left(i\right)h\left(i\right)y\left(i\right)\right], \end{array}$$

therefore $\hat{\theta }(k)=\hat{\theta }(k-l)+P(k)H_{k,l}^{T}\Lambda_{k,l}[y_{k,l}-H_{k,l}\hat{\theta }(k-l)]$, and assume that *K*(*k*) = *P*(*k*)*H*_*k*,*l*_^*T*^Λ_*k*,*l*_, then there is

(10) $$\begin{array}{c} \hat{\theta }\left(k\right)=\hat{\theta }\left(k-l\right)+K\left(k\right)\left[y_{k,l}-H_{k,l}\hat{\theta }\left(k-l\right)\right]. \end{array}$$

According to the matrix inversion formula, there is

(11) P(k)=P(k−l){I−Hk,lT[Hk,lP(k−l)Hk,lT         +Λk,l−1]−1Hk,lP(k−l)},K(k)=P(k−l)Hk,lT[Hk,lP(k−l)Hk,lT+Λk,l−1]−1.

According to (10) and (11), the variable step-size recursive least square estimation algorithm can be derived

(12) $$\begin{array}{c} K\left(k\right)=P\left(k-l\right)H_{k,l}^{T}{\left[H_{k,l}P\left(k-l\right)H_{k,l}^{T}+\Lambda_{k,l}^{-1}\right]}^{-1},\\ P\left(k\right)=\left[I-K\left(k\right)H_{k,l}\right]P\left(k-l\right),\\ \hat{\theta }\left(k\right)=\hat{\theta }\left(k-l\right)+K\left(k\right)\left[y_{k,l}-H_{k,l}\hat{\theta }\left(k-l\right)\right]. \end{array}$$

## 4. Simulation Study

In this section, simulation experiments are investigated to verify the validity of the proposed model method with different identification methods.

**Figure 5:**
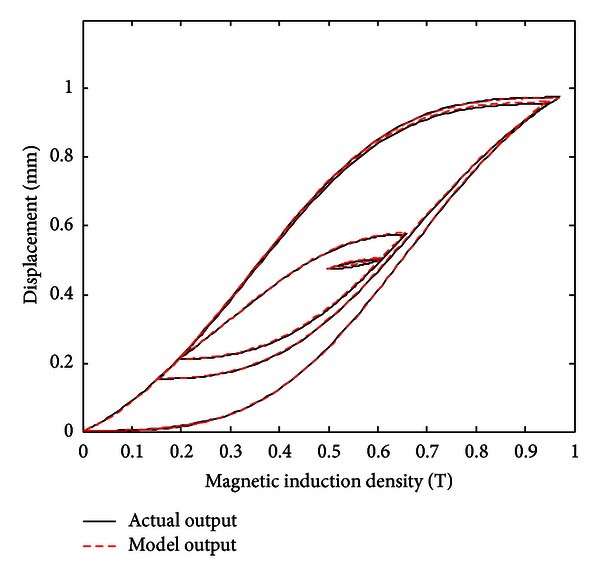
Comparison of actual hysteresis loop and simulation hysteresis loop (with the improved gradient correction algorithm).

**Figure 6:**
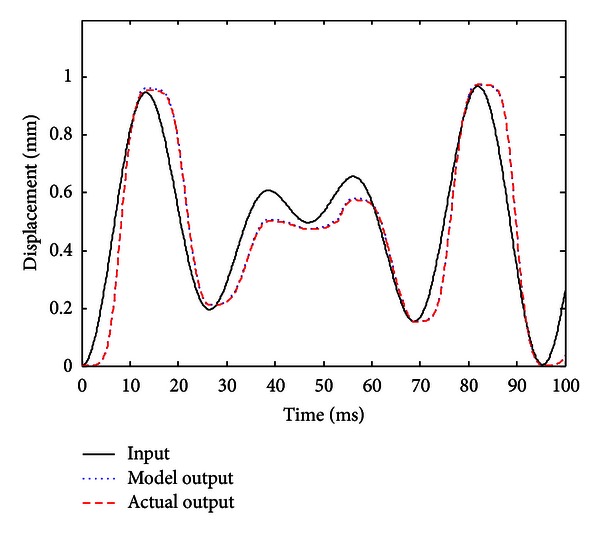
Actual output curve and model output curve (with the improved gradient correction algorithm).

**Figure 7:**
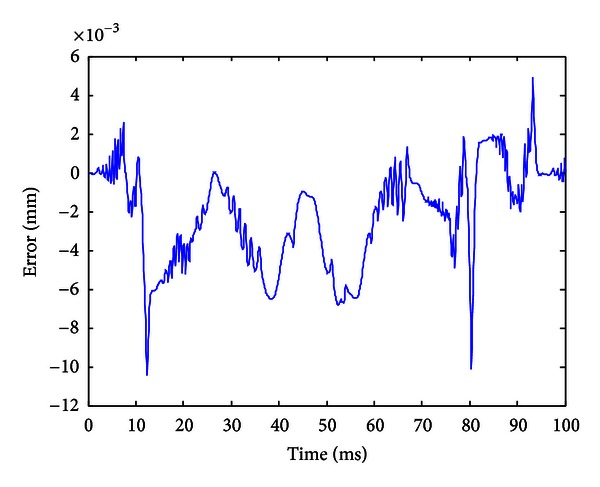
Modeling error (with the improved gradient correction algorithm).

When the improved gradient correction algorithm is adopted and the number of KP model's discretization lines *L* is set to *L* = 15, the expectation error *E* is set to *E* = 0.01, the maximum cycle number MCN = 50, and the simulation result is shown in Figures 5, 6, and 7.  Figure 5 shows the comparison of actual hysteresis loop and simulation hysteresis loop, Figure 6 shows the actual output and model output of MSMA actuator, and Figure 7 shows the modeling error.

From the simulation results, it can be seen that the proposed KP model with improved gradient correction algorithm can be used to establish a good hysteresis model which has the ability to describe both the major hysteresis loop and inner hysteresis loops, and the maximum modeling error is 0.0104 mm.

**Figure 8:**
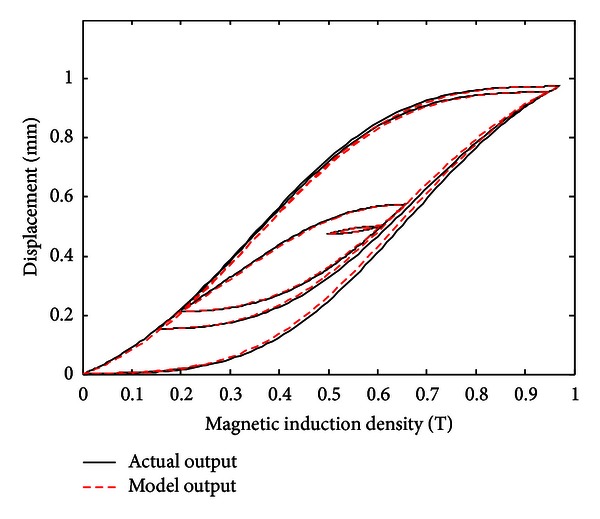
Comparison of actual hysteresis loop and simulation hysteresis loop (with the variable step-size recursive least square estimation algorithm).

**Figure 9:**
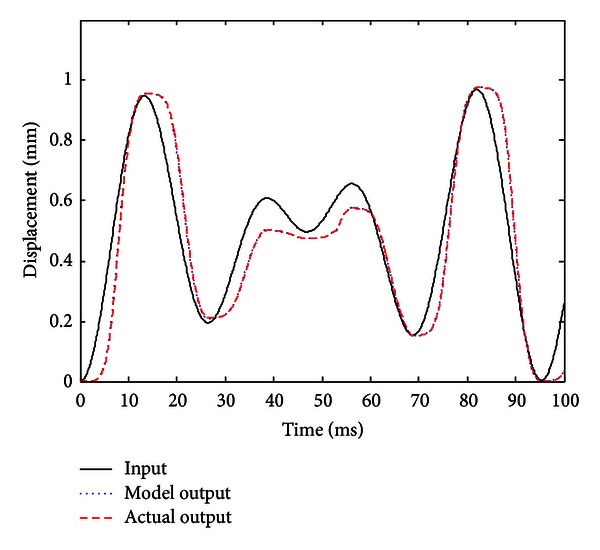
Actual output curve and model output curve (with the variable step-size recursive least square estimation algorithm).

**Figure 10:**
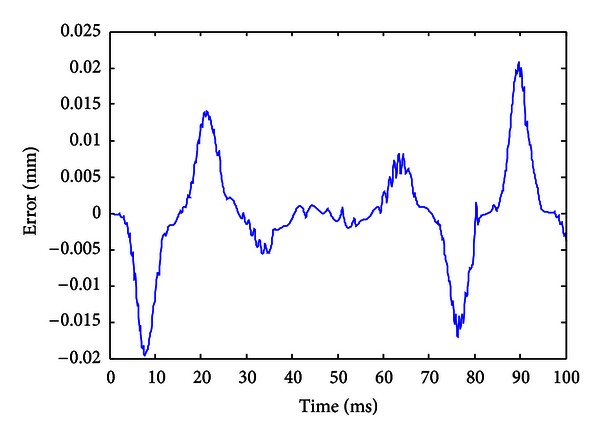
Modeling error (with the variable step-size recursive least square estimation algorithm).

When the variable step-size recursive least square estimation algorithm is adopted and the number of KP model's discretization lines *L* is set to *L* = 15, the step-size of the variable step-size recursive least square estimation algorithm is set to *l* = 3 and then the simulation result is shown in Figures 8, 9, and 10.

The simulation results show that with the variable step-size recursive least square estimation algorithm, the established hysteresis model can also have a good performance in describing the hysteresis between input and output of MSMA actuator, and the maximum modeling error is 0.0209 mm.

In the simulation experiments above, there are some optional parameters as the number of KP model's discretization lines *L*, expectation error *E* and the maximum cycle number MCN in the improved gradient correction algorithm, and the step-size *l* in the variable step-size recursive least square estimation algorithm. According to the mathematical principles above, the modeling accuracy can be further improved by changing these optional parameters.

**Figure 11:**
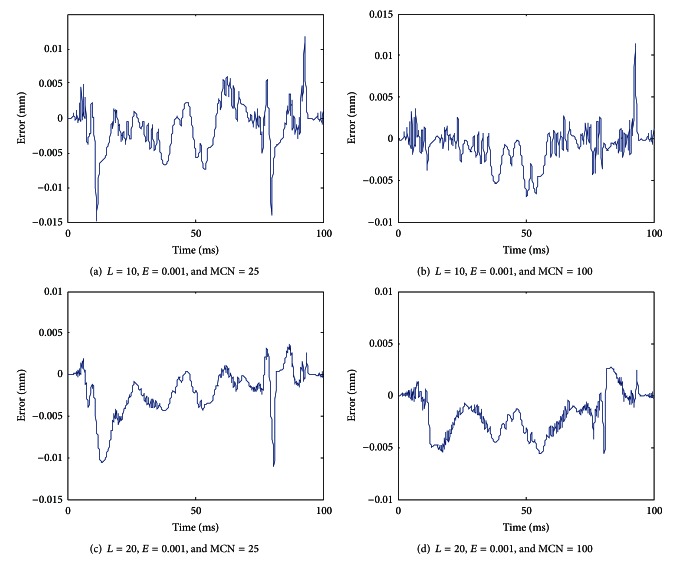
Modeling error when the optional parameters have different values (with the improved gradient correction algorithm).

With the improved gradient correction algorithm, Figure 11 shows the modeling error when the optional parameters have different values.

From Figure 11, it can be seen that by increasing the number of discretization lines *L* and the maximum cycle number MCN, the modeling error can be reduced. When *L* = 20, *E* = 0.001, and MCN = 100, as shown in Figure 11(d), the maximum modeling error can be reduced to 0.0056 mm.

**Figure 12:**
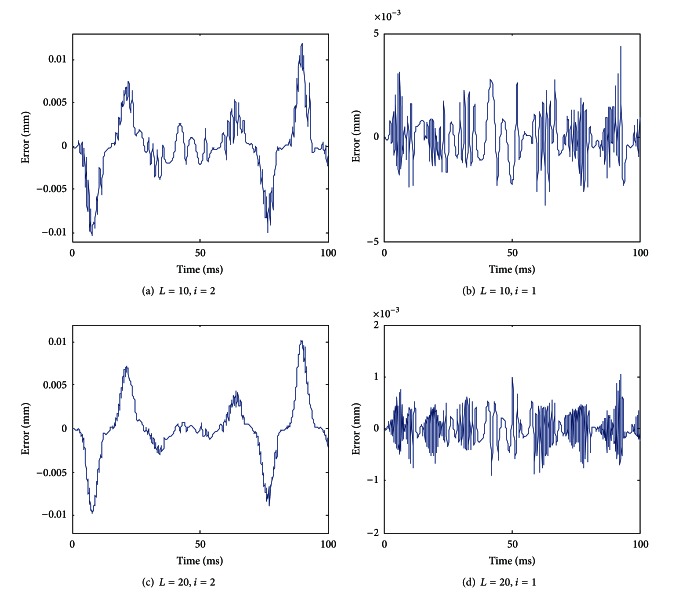
Modeling error when the optional parameters have different values (with the variable step-size recursive least square estimation algorithm).

With the variable step-size recursive least square estimation algorithm, Figure 12 shows the modeling error when the optional parameters have different values.

Just as expected, Figure 12 shows that when the number of discretization lines *L* is increased or the step-size *l* is reduced, the modeling error is reduced. When *L* = 20 and *l* = 1, the maximum modeling error can be reduced to 0.001 mm.

The experiment results in this section can verify the validity of the proposed hysteresis model in this paper, and from the comparison of the simulation results of the two identification algorithms, it can be seen that the variable step-size recursive least square estimation algorithm has a better performance in the identification, and the modeling accuracy can be adjusted by adjusting the optional parameters in the proposed model.

## 5. Conclusion

A mathematic modeling method based on KP model is proposed to describe the hysteresis of the MSMA actuator in this paper. In order to identify the weighting parameters in the KP model, an improved gradient correction algorithm and a variable step-size recursive least square estimation algorithm are adopted, and the simulation results have proven the validity of the proposed hysteresis model. By adjusting the optional parameters of this hysteresis model, the modeling accuracy can be further improved, and in the simulation experiment, the modeling error can be reduced to 0.001 mm with the variable step-size recursive least square estimation algorithm. A satisfactory hysteresis model can be gotten if the optional parameters are adjusted according as the requirements of modeling accuracy and computational complexity.
